# Step-wise endoscopic approach to palliative bilateral biliary drainage for unresectable advanced malignant hilar obstruction

**DOI:** 10.1038/s41598-019-48384-x

**Published:** 2019-09-13

**Authors:** Jin Ho Choi, Sang Hyub Lee, Min Su You, Bang-sup Shin, Young Hoon Choi, Jinwoo Kang, Sunguk Jang, Woo Hyun Paik, Ji Kon Ryu, Yong-Tae Kim

**Affiliations:** 1 Department of Internal Medicine, Liver Research Institute, Seoul National University College of Medicine, Seoul, Korea; 2 Department of Gastroenterology and Hepatology, Cleveland Clinic, Cleveland, USA

**Keywords:** Bile duct cancer, Gastrointestinal diseases

## Abstract

The ideal type of stent utilized at index endoscopic retrograde cholangiopancreatography (ERCP) in management of malignant hilar obstruction (MHO) remains unclear. We aimed to determine the ideal stent choice in patients with MHO. In this retrospective study, patients with unresectable MHO were separated into the plastic stent (PS) group and the self-expandable metal stent (SEMS) group. The primary outcome was the risk and rate of rescue percutaneous transhepatic biliary drainage (PTBD). The secondary outcomes were the progression-free survival, the overall survival and the PTBD-free period (days). Thirty-six patients in the PS group and 38 patients in the SEMS group were enrolled. The risk for PTBD was higher in SEMS group (HR = 2.205, 95% C.I. 0.977–4.977, P = 0.057). The rate of PTBD was significantly lower in the PS group. (22.2% vs 50.0%, P = 0.017) There were no differences in overall survival and progression-free survival (410 and 269 in the PS group, 395 and 266 in the SEMS group, P = 0.663 and P = 0.757). The PTBD-free period was significantly longer in the PS group. (836.43 vs 586.40, P = 0.039) Although comparable in clinical efficacy, utilization of PS at index ERCP may reduce patient’s discomfort by avoiding PTBD and prolonging PTBD-free period in patients with MHO.

## Introduction

Patients with malignant hilar obstruction (MHO) from biliary tract cancer have poor prognosis as only 20–30% of the patients are amenable for surgical resection^1,2^. Unresectable MHO is managed with non-surgical treatments including endoscopic or percutaneous biliary drainage, chemo and, radiotherapy, which can improve the quality of life (QOL) and length of survival (LOS)^3,4^. At present, endoscopic retrograde cholangiopancreatography (ERCP) with stent placement is the standard treatment for biliary drainage in the patients with MHO^5^.

Among several controversies in regards to ERCP in patients with MHO, the type of stent chosen at index ERCP remains a point of healthy debate. Plastic stent (PS) has low unit cost, is easier to place and revise when necessary. On the other hand, it has poor patency duration and higher migration rate compared to self-expandable metal stent (SEMS)^6,7^. Due to larges luminal diameter with stronger radial force, SEMS has shown better efficacy in biliary drainage, longer patency duration, less frequent need for revision, and longer survival length^7–10^. Its disadvantages include its cost and technical difficulty. Thus, it is often recommended to place PS if the life expectancy is less than 3 months whereas SEMS should be use in patients whose life expectancy is longer than 3 months^2^.

To date, few studies exist regarding optimal stent for unresectable MHO based on reduction of endoscopic or percutaneous re-intervention when initial endoscopic retrograde biliary drainage (ERBD) succumbs to restenosis. As recent advancements in palliative therapies have resulted in improved longevity for the patients with unresectable MHO, it is logical to assume that the frequency of endoscopic or percutaneous revision of restenosis has also increased in recent years^11–15^. Indeed, many patients with unresectable MHO with good performance may “outlast” the expected patency duration of SEMS requiring stent revision or replacement. The success rate of the endoscopic revision for bilateral SEMS is reported as 40–92% in previous studies^10,16–19^. Although there may be no difference in the success rates of primary revision between PS and SEMS after re-occlusion of index ERBD, repeated revision with SEMS is often more difficult than repeated PS replacement and will eventually rely on percutaneous transhepatic biliary drainage (PTBD) more frequently than revision with PS. As such, it is important to ascertain the optimal type of stent at index ERBD, the difficulties of endoscopic revision and PTBD ought to be avoided as they may increase patient’s discomfort.

In this study, we aimed to compare clinical efficacy, survival and patency durations and the need for revision due to re-occlusion after of initial ERBD based on the type of stent (PS or SEMS) used at the time of index ERBD.

## Methods

### Study design and patients

This retrospective cohort study was conducted with the patients diagnosed with unresectable MHO from cholangiocarcinoma or gallbladder cancer (Bismuth type III or IV)^20^ at Seoul National University Hospital from January 2010 to December 2016. Only the patients with histologic confirmation of aforementioned primary biliary tract malignancies were included. Other inclusion criteria consist of 1) adult (20 or older) patients with good functional performance status defined by the Eastern Cooperative Oncology Group (ECOG) scale of 2 or less, and 2) the patients with planned palliative treatment (either chemo, radio or photodynamic therapy) for non-surgical treatment option. The patients with previous percutaneous biliary drainage prior to index ERBD, poor functional performance (ECOG scale greater than 2) or follow up duration less than 3 months were excluded. Patients with Bismuth II obstruction were excluded because it may be sufficient for unilateral drainage in some cases^2^, and endoscopic revision for bilateral stent is relatively less demand for the technical skills compared to higher grade MHO.

Patients were divided into two groups based on the type of stent used (PS or SEMS) at index endoscopic biliary drainage immediately following pathologic confirmation of biliary tract malignancy as the cause of their hilar stricture. The pre-intervention data including age, sex, serum laboratory data, functional performance status (ECOG scale), and Charlson comorbidity index (CCI) were collected. From endoscopic reports, details of stricture type (Bismuth classification), length, stent type (PS or SEMS) and fluoroscopic and endoscopic appearances of post stent deployment were gathered. Electronic and archived paper medical records were used for the patient follow up to assess clinical success, length of survival and need for re-intervention. This study was approved by the institutional review board of the Seoul National University Hospital, Korea (IRB No. H-1707-083-870) and all methods were carried out in accordance with relevant guidelines and regulations. Due to the retrospective design of the study, the informed consent was waived.

### Outcomes and definitions

#### Study outcomes

The primary outcome was comparison of the risk and rate of rescue PTBD for failed endoscopic revision of ERBD in both groups. The secondary outcomes were progression free survival (PFS), overall survival (OS), the number of endoscopic ERBD revision, and the PTBD-free period during follow-up.

#### Definitions

Revision of ERBD: Endoscopic revision and rescue PTBD: Patients in the both group were assessed for endoscopic revision with either PS or SEMS in the event of restenosis during follow-up. Endoscopic revision was the first choice and the its timing was determined based on the clinical signs and symptoms of recurrent obstruction such as acute cholangitis (exacerbation of jaundice, abdominal pain, and fever >38’C), inflammatory response (leukocytosis <4 or >10 × 1,000/uL, c-reactive protein ≥1 mg/dL), abnormal liver function tests (alkaline phosphatase, r-glutamyltransferase, alanine aminotransferase, or aspartate aminotransferase >1.5 × upper normal limit) or exacerbation of jaundice even without symptoms. Rescue PTBD was performed in patients who technically or clinically failed endoscopic revision and who had fatal clinical conditions including shock state or bleeding. Sessions for photodynamic therapy (PDT) were not counted as number of ERBD revision. The stent patency was defined as the interval between the time of stent placement and that of first stent dysfunction. If no stent dysfunction was apparent during follow-up, the stent patency was regarded as duration from the time of stent insertion to patient death or last hospital visit.

Technical and clinical success: Technical success was defined as the ability to access and drain through hilar obstruction by bilateral stent placement. Clinical success was defined as complete resolution of symptoms with reduction in serum bilirubin to normal value (≤1.2 mg/dL) or less than half of the pretreatment level within 2 weeks. These criteria were also applied to endoscopic revision.

Survival and PTBD-free period: PFS (days) was defined as the interval between the time of diagnosis to either the time when clinical evidence of disease progression was noted or end of follow-up for censored cases. OS (days) was defined as the duration between the time of diagnosis and that of death or last follow-up date. The PTBD-free period referred to the duration (days) until the first PTBD was performed, or the duration (days) until the last follow-up if PTBD was not performed.

### Details of procedure

ERBD was performed with therapeutic duodenoscopy (TJF-260v, JF-260v, TJF-240, JF-240, Olympus, Tokyo) by 4 gastroenterologists. Plastic biliary stents which were used in initial biliary drainage and endoscopic revision were as follows: Cotton-Leung ® (Cook Medical, Inc, Bloomington, IN), Advanix™ duodenal bend (Boston Scientific, Natick, MA, USA), Zimmon® (Cook Medical, Inc, Bloomington, IN), and Advanix™ double pigtail (Boston Scientific, Natick, MA, USA). SEMS which were used in this study were as follows: BONASTENT® (Standard Sci-Tech, Seoul, Korea), Niti-S™ Biliary Stent (D-type and LCD type) (Taewoong medical, Seoul, Korea), Zilver 635® (Cook Medical, Inc, Bloomington, IN), and WallFlex™ (Boston Scientific, Natick, MA, USA). All procedures were performed by four experienced and skilled endoscopists with more than 500 endoscopic retrograde cholangiopancreatography (ERCP) procedures per year.

### Statistical analysis

Continuous variables were analyzed by the Student’s t-test and categorical variables were analyzed using the chi-squared test or Fisher’s exact test. P values less than 0.05 were considered significant. For the risk and rate of rescue PTBD, we performed competing risk analysis treating death without rescue PTBD as a competing risk to present hazard ratio and cumulative incidence functions plot. Information about death was recognized if the patient has died in our institution or if the medical record contained a mention of death in the medical record. Survival and duration of PTBD-free period was analyzed using the Kaplan–Meier method and log-rank test. The cox-proportional hazard model was used to analyze the factors affecting PTBD-free period. All statistical analyses were performed using SPSS v.23.0 (IBM Corp, Armonk, NY, USA) and SAS version 9.4 (SAS Institute, Inc., Cary, NC, USA).

## Results

### Baseline characteristics

**Figure 1:**
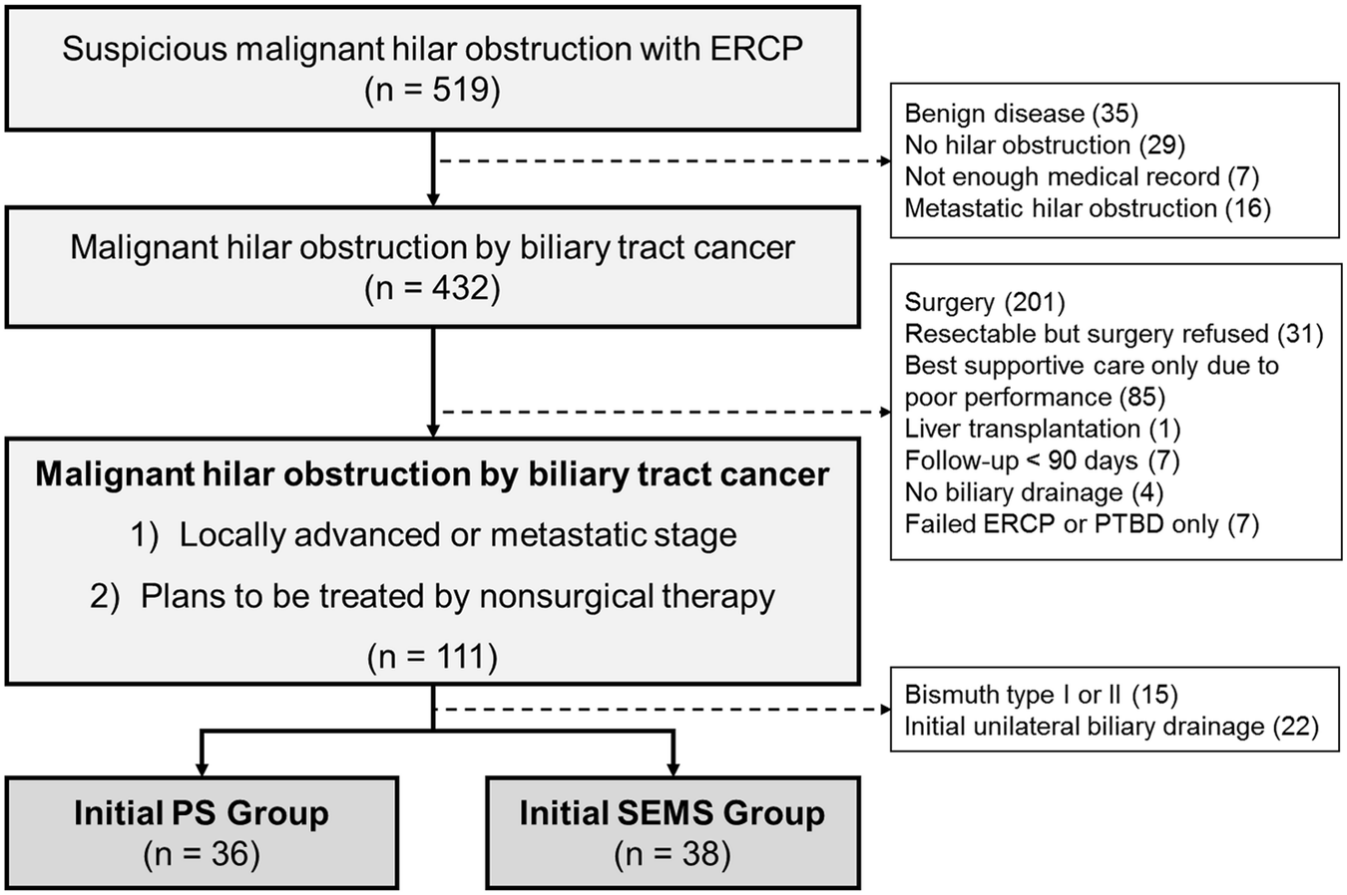
Flowchart of this study. PS, plastic stent; SEMS, self expendable metal stent; PTBD, percutaneous transhepatic biliary drainage; ERBD, endoscopic retrograde biliary drainage.

A total of 519 patients were underwent ERCP for the management of MHO, of which 432 patients were diagnosed with primary biliary tract malignancy (Fig. 1). Of 432 subjects, additional 321 patients were excluded due to various reasons including eventual surgical resection (201 patients), poor performance status (85 patients) refusal of surgery (31 patients), follow up duration less than 90 days (7 patients) and ERCP failure (7 patients). From remaining 111 patients, another 37 patients were excluded due to Bismuth classification of I or II (15 patients) and failure of bilateral drainage (22 patients), leaving 36 patients in the PS group and 38 patients in the SEMS group. (Fig. 1).

The baseline characteristics of the study patients are shown in Table 1. With the exception of the type of biliary tract cancer (34 cholangiocarcinoma and 2 gallbladder cancer in PS group versus 29 cholangiocarcinoma and 9 gallbladder cancer in SEMS group, P = 0.047), there were no significant differences in sex, age, performance, comorbidity, stage, and the Bismuth type between the two groups. The mean follow-up duration (month) of PS group was 15.06 ± 7.92 and 16.42 ± 13.49 for SEMS group (P = 0.601). In terms of treatment, 91.9% (68 of 74) was treated with palliative cancer therapy including chemotherapy (64 patients), radiation therapy (3), and PDT (13). Six patients who were initially planned chemotherapy received supportive care only due to patients’ refusal.

**Table 1:** Baseline characteristics.

		PS group (n = 36)	SEMS group (n = 38)	P
Sex	Male	24 (66.7%)	23 (60.5%)	0.635
	Female	12 (33.3%)	15 (39.5%)	
Age (years)	66.9 ± 11.0	67.5 ± 7.4	0.790	
ECOG	0, 1	32 (88.9%)	35 (92.1%)	0.707
	2	4 (11.1%)	3 (7.9%)	
Comorbidity (Charlson’s comorbidity index)		8.86 ± 1.6	9.18 ± 1.7	0.407
Diagnosis	Cholangiocarcinoma	34 (94.4%)	29 (76.3%)	0.047
	Gallbladder cancer	2 (5.6%)	9 (23.7%)	
Stage	Locally advanced	21 (58.3%)	13 (34.2%)	0.061
	Distant metastasis	15 (41.7%)	25 (65.8%)	
Bismuth type	III	10 (27.8%)	14 (36.8%)	0.462
	IV	26 (72.2%)	24 (63.2%)	
Follow-up duration, mean ± SD (month)		15.06 ± 7.92	16.42 ± 13.49	0.601

### Evaluation of primary and secondary outcomes

**Figure 2:**
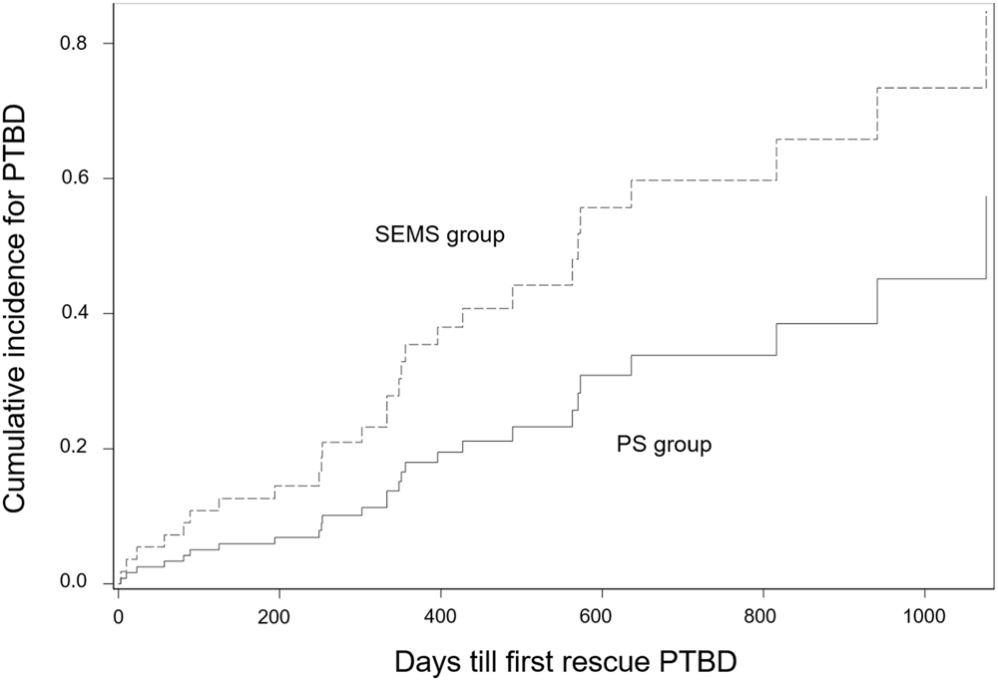
The cumulative incidence functions plot of the risk for rescue PTBD in both group. PS, plastic stent; SEMS, self expendable metal stent; PTBD, percutaneous transhepatic biliary.

**Figure 3:**
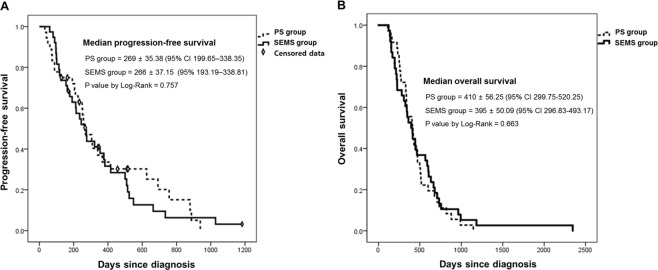
Progression-free survival and overall survival according to initial drainage method. PS, plastic stent; SEMS, self expendable metal stent; PTBD, percutaneous transhepatic biliary drainage; ERBD, endoscopic retrograde biliary drainage.

**Figure 4:**
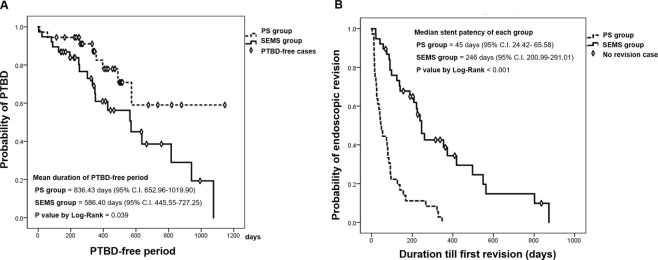
The analysis for the PTBD-free period and stent patency in both groups. PS, plastic stent; SEMS, self expendable metal stent; PTBD, percutaneous transhepatic biliary drainage; ERBD, endoscopic retrograde biliary drainage.

The risk for rescue PTBD was higher in SEMS group than PS group but no statistical significance was observed. (Hazard ratio = 2.205, 95% C.I. 0.977–4.977, P = 0.057). The cumulative incidence functions plot was shown in Fig. 2. The rate of rescue PTBD was significantly lower in the PS group than the SEMS group. (22.2% vs 50.0%, P = 0.017) (Table 2). The causes of PTBD was endoscopic revision failure or fatal clinical condition, which were 62.5% and 37.5% in the PS group and 84.2% and 15.8% in the SEMS group, respectively. The median PFS (days) was 269 ± 35.38 (95% C.I. 199.65–338.35) in the PS group and 266 ± 37.15 (95% C.I. 193.19–338.81) in the SEMS group. (P = 0.757) (Fig. 3A). The median OS (days) was 410 ± 56.25 (95% C.I. 299.75–520.25) in the PS group and 395 ± 50.09 (95% C.I. 296.83–493.17) in the SEMS group. (P = 0.663) (Fig. 3B). The analysis for the PTBD-free period in the both groups were presented with Kaplan-Meier plot, and we presented the mean value rather than median value because PTBD was inserted in less than 50% of the PS group. (Fig. 4A). The mean duration of PTBD-free period (days) were 836.43 ± 93.61 (95% CI 652.96–1019.90) for PS group and 586.40 ± 71.86 (95% CI 445.55–727.25) for SEMS group. (P = 0.039) The mean number of ERBD revision was significantly higher in the PS group than the SEMS group. (4.14 ± 2.54 vs 1.68 ± 1.58, P < 0.001).

**Table 2:** Comparison of the biliary drainage patterns and the revision profile of both groups.

				PS group (n = 36)	SEMS group (n = 38)	P
Initial drainage			Technical success	36 (100%)	38 (100%)	
			Clinical success	35 (97.2%)	37 (97.4%)	
Bilateral SEMS method			Stent-in-stent	NA	26 (68.4%)	
			Stent-by-stent	NA	12 (31.6%)	
Risk for rescue PTBD				1	2.205 (95% CI 0.977–4.977)	0.057
Rate of rescue PTBD				8 (22.2%)	19 (50.0%)	0.017
Reason for rescue PTBD						
Endoscopic revision failure				5 (62.5%)	16 (84.2%)	
Fatal clinical conditions				3 (37.5%)	3 (15.8%)	
Conversion to SEMS				19 (52.8%)	NA	
Duration till conversion to SEMS (days), mean ± SD				98.1 ± 143.0	NA	
Number of ERBD revision, mean ± SD				4.14 ± 2.54	1.68 ± 1.58	<0.001
Revision needed cases				36 (100.0%)	28 (73.7%)	0.001
Entire endoscopic revision	Technical success	No SEMS (n = 17)	12 (70.6%)	No revision (n = 8)	NA	
		SEMS (n = 19)	14 (73.7%)	Revision (n = 28)	12 (31.6%)	
	Clinical success	No SEMS (n = 17)	16 (94.1%)	No revision (n = 8)	NA	
		SEMS (n = 19)	12 (63.2%)	Revision (n = 28)	17 (44.7%)	
Revision after SEMS conversion	None	6		NA		
	1	6		NA		
	≥2	7		NA		
Duration of PTBD maintenance (days), mean ± SD		35.0 ± 118.3		70.6 ± 167.4		0.296

*PS, plastic stent; SEMS, self expendable metal stent; PTBD, percutaneous transhepatic biliary drainage; ERBD, endoscopic retrograde biliary drainage; SD, standard deviation; NA, not applicable; CI, confidence interval.

### Details of the other features of the biliary drainage

Among PS group, 27.8% of patients experienced technical failure of endoscopic revision and 22.2% experienced clinical failure from endoscopic revision. The clinical success rates of initial endoscopic drainage showed no significant statistical difference. (97.2% in the PS group versus 97.45% in the SEMS group) All patients in the PS group eventually required ERBD revision during the follow up period while 73.7% in the SEMS group required revision of their index ERBD during follow-up.(P = 0.001). The mean number of ERBD revision was higher in the PS group than the SEMS group. (4.14 ± 2.54 vs 1.68 ± 1.58, P < 0.001) The median stent patency duration was shown in Fig. 4B and significantly longer in the SEMS group than the PS group. (246 days (95% CI 200.99–291.012) vs 45 days (95% CI 24.42–65.58), P < 0.001). Among the PS group, 52.8% (19 of 36) patients underwent conversion to SEMS, and the mean interval from index PS placement to SEMS conversion was 98.1 ± 143.0 days. After SEMS conversion, 68.4% (13 of 19) patients underwent ERBD revision, with and 2 or more ERBD revisions were performed in 36.8% patients. There was no technical failure or clinical failure of 1^st^ ERBD revision for PS and the rates of technical and clinical failure of 2^nd^ ERBD revision for PS were 8.33% and 2.78%, respectively. Technical failure of endoscopic revision in the PS group mostly occurred during revision for SEMS except 1 patient. Among the patients who were needed endoscopic revision in the SEMS group, 31.6% patients were experienced technical failure of endoscopic revision and 44.7% patients were experienced clinical failure of endoscopic revision. The technical and clinical failure of 1^st^ ERBD revision for SEMS were 8.78% and 19.3%, respectively.

Among the subjects in the PS group who received PS for endoscopic revision (n = 17), 70.6% of them had technical success with 94.1% of clinical success rate. One patient (5.9%) underwent rescue PTBD. Among the subject that were converted to SEMS during their endoscopic revision (n = 19), the rates of technical success and clinical success were 73.7% and 63.2% respectively. Seven patients (36.8%) underwent rescue PTBD. There was significant difference in rate of rescue PTBD between patients with and without SEMS conversion in PS group. (P = 0.026) A total of 71.4% (5 of 7) patients who underwent PTBD among the patients of the PS group with SEMS conversion were performed rescue PTBD after SEMS conversion.

Cox proportional analysis revealed the choice of stent at the index endoscopic biliary drainage exhibited statistically significant effect on PTBD-free period in favor of PS in univariate analysis (Hazard ratio = 2.343, 95% C.I 1.020–5.383, P = 0.045) and multivariate analysis (Hazard ratio = 2.533, 95% C.I 1.045–6.236, P = 0.040). Whereas, other factors such as cancer type, bismuth type, stage, performance status, treatment modality did not affect PTBD-free period. (Table 3).

**Table 3:** Results of cox-proportional analysis for factors affecting PTBD-free period.

Covariate*	Univariate		Multivariate	
	HR (95% CI)	P-value	HR (95% CI)	P-value
Sex	1.364 (0.636–2.924)	0.425		
Age	0.676 (0.301–1.516)	0.342		
Type of cancer	1.434 (0.415–4.959)	0.569	0.919 (0.234–3.601)	0.903
Performance status	1.917 (0.432–8.494)	0.392	2.033 (0.442–9.339)	0.362
Stage	1.237 (0.566–2.704)	0.594	0.952 (0.402–2.254)	0.911
Bismuth type	0.710 (0.309–1.632)	0.420	0.826 (0.536–1.274)	0.826
Stent type	2.343 (1.020–5.383)	0.045	2.553 (1.045–6.236)	0.040
Photodynamic therapy	0.249 (0.058–1.061)	0.060		
Chemotherapy	1.219 (0.285–5.218)	0.790		
Radiotherapy	0.598 (0.080–4.466)	0.616		

PS, plastic stent; SEMS, self expendable metal stent; PTBD, percutaneous transhepatic biliary drainage; HR, hazard ratio; CI, confidence interval.

*The comparison factor of each covariance is as follows, and it is set as the latter reference value. Sex (female, male), Age (over than median, lower than median, Type of cancer (gallbladder cancer, cholangiocarcinoma), Performance status (ECOG 2, ECOG 0 or 1), Stage (stage IV, stage III), Bismuth type (Bismuth IV, Bismuth III), Stent type (SEMS, PS), Photodynamic therapy (done, not done), Chemotherapy (done, not done), Radiotherapy (done, not done).

The duration of PTBD maintenance were 35.0 ± 118.3 (range, 14–615) days in PS group and 70.6 ± 167.4 (range, 6–841) days in SEMS group (P = 0.296). The removal of PTBD were possible in 25.0% of patients in the PS group and 10.5% of patients in the SEMS group. (P = 0.333).

The method of bilateral stenting (stent-in-stent vs stent-by-stent) in the SEMS group did not produce any significant differences in the rate of rescue PTBD, median PFS, OS, PTBD-free duration, or total number of ERBD revision sessions.

## Discussion

To our knowledge, this is first study reporting benefit of PS over SEMS in management of MHO which considering patient discomfort by rescue PTBD and the period that initial percutaneous drainage might be avoided. The majority similar prior studies have focused on comparative clinical success rates, stent patency duration or survival durations among rather heterogeneous patient population^7,9,10,21,22^. We observed in our practice a steady increase in number of ERBD revision among the patients with unresectable MHO as their length of survival has seen steady rise in recent years. It can certainly predisposes such patients to increased need of biliary drainage revision over time, hence increasing the risk of eventual failure of endoscopic drainage and subsequent reliance on PTBD. As percutaneous biliary drainage not only predisposes the patients with biliary obstruction to increased patient’s discomfort in everyday life due to tube related problems and the necessity of tube management, judicious selection of endoscopic stent should sought to reduce the risk of endoscopic technical failure resulting in reliance to percutaneous drainage. To that end, our study showed that PS stent affords higher resilience of endoscopic revision and a lower risk and rate of faltering to percutaneous rescue therapy with delay of the period until initial PTBD. Although the hazard ratio was shown no statistical significance at the 5% level of bilateral test by competing risk analysis, the results of this study seems to be clinically meaningful because the difference in cumulative incidence over time is evident despite the relatively small number of cases.

**Figure 5:**
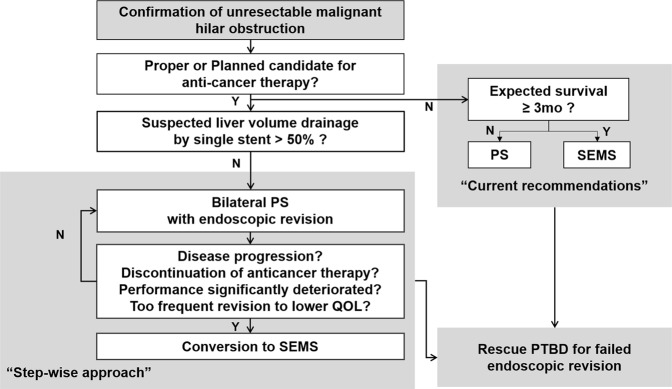
The proposed algorithm of the stent type for palliative bilateral biliary drainage for unresectable advanced malignant hilar obstruction: A step-up approach. PS, plastic stent; SEMS, self expendable metal stent; PTBD, percutaneous transhepatic biliary drainage; ERBD, endoscopic retrograde biliary drainage.

Although the SEMS group required lower number of endoscopic revisions stemming from due to longer stent patency when restenosis occurred, the success rate of repeat endoscopic stent placement was significantly lower in SEMS than the PS groups, resulting in a higher rate of PTBD reliance. In addition, no survival benefit was observed in SEMS compared to the PS group. In comparison with PS but they did not show survival gain than the PS group. A plausible explanation for the lower success rate of endoscopic revision among the SEMS group is that reactive tissue hyperplasia surrounding metal stent is more robust than that of PS stent, resulting in propagation of stenotic area within and above the SEMS. Based on our new findings and comparable lengths of PFS and OS observed from the previous study^14^, we propose an algorithm of step-wise approach for the palliative bilateral biliary drainage for unresectable advanced malignant hilar obstruction in Fig. 5. It is recommended to insert SEMS in all patients in this study due to the longer expected survival over 3 months according to current guideline^2^, but the patients in PS group were managed according to this step-wise approach.

The median lengths of PFS and OS observed in our study were longer than the known life expectancy in similar previous studies^9,10,19,23^. Our study showed a similar PFS and better OS, as compared to that reported in previous clinical trials of chemotherapy^14^. This can be attributed to the selection criteria of our study that were biased toward the patients with good performance status (ECOG scale of 2 or less), as well as toward those with successful bilateral stenting only which is a known factor of longer overall survival. There remains a controversy regarding differences in survival benefit between PS and SEMS in MHO. A randomized control trial by Mukai and colleagues reported findings similar to ours which showed no significant difference in the length of OS between the PS and SEMS^10^. In comparison, Sangchan and colleagues in their randomized control trial of 108 patients reported a significantly longer survival duration with SEMS use than PS^9^. Both studies showed a shorter survival period compared to our study because of the lower proportion of patients who received anticancer therapy and shorter follow-up periods. We believe there is no significant benefit in the length of survival based on the type of stent chosen when managing the patients with MHO whose performance status and treatment of cancer treatment favorable, if active managements are taken against to cholangitis.

The overall technical and clinical success rates of endoscopic revision in this study are lower than that of previously reported. This is likely due to our inclusion of all endoscopic revision (including all repeat revision after the primary revision) whereas other studies only included first revision after index ERCP. As technical complexity increases with repeated endoscopic manipulations of the same stricture site, this could in turn increase risk of technical and clinical failure. Indeed, when the subgroup of primary revision was analyzed, the success rates in our studies were comparable with previous studies^10,17–19,23^.

When considering the aspect of patient’s discomfort in patients with MHO, we believe avoidance or delay of percutaneous drainage is of a paramount importance due to aforementioned potential adverse outcomes related to PTBD. In that regard, our study demonstrated longer PTBD-free period as well as lower rate of rescue PTBD in the PS group than SEMS group support our belief that PS ought to be considered as initial choice of stent in MHO. In fact, PTBD was rarely needed in the PS group without conversion to SEMS.

This study has several limitations. First, this was a retrospective study. As such, the absence of pre-formed protocol introduced significant inherent heterogeneity in decision making and data collection. For example, decision to choose one type of stent over the other was based on individual physician’s clinical decision (i.e. not randomly assigned) and the decision of conversion to SEMS after initial PS was made without clear criteria. In our institution, consistent criteria for the type of initial stent was not specified, although almost all patients who undergo PDT were inserted PS and SEMS was usually chosen in case of no possibility of surgery by reducing tumor burden despite anticancer therapy. Meanwhile, the conversion to SEMS was mainly performed as the proposed algorithm (Fig. 5), which is based on consideration of mentioned clinical conditions including disease progression, discontinuation of anticancer therapy, performance deterioration, or too frequent revision to lower quality of life. Second, the small sample size of this study could introduce type I error (false rejection of true null-hypothesis). Third, although we assumed that patients with PTBD may have more discomfort based on our experience, we decided to suggest PTBD-free duration as a surrogate marker of patient’s discomfort because we thought that PTBD-related problems such as pain, tube management (disinfection, exchange, etc.), tube retraction, and exit site infection puts more burden on the patient than multiple endoscopic revisions. However, the exact causal relationship between patient’s discomfort and PTBD was could not investigated because of the limitations of research design and lack of quantitative analysis of QOL. It would be better to perform a cost-effectiveness analysis to evaluate the effeciency on the patient, but it was difficult to conduct with detailed information in this retrospective study. Finally, there was a difference in the ratio of gallbladder cancer and cholangiocarcinoma in the both groups. This could possibly introduce treatment bias. As there was higher prevalence of cholangiocarcinoma patients in PS group there could also be higher prevalence of patients who undergo PDT for cholangiocarcinoma in PS groups than SEMS group.

There are several strengths of this study. First, as we know, this is the first study to compare PS and SEMS by reflecting more realistic aspects in implementing palliative ERBD for unresectable MHO, which not only focused on the technical aspects of procedures. Second, this study was conducted on the patients who had a relatively good prognosis and had a great demand for the procedure and whose role was important. If the palliative treatment for unresectable MHO is further developed in the future, this study is expected to have more significance because the number of patients with setting similar to the patients of this study will increase.

In conclusion, our study demonstrated lower likelihood of endoscopic treatment failure with PS which in turn resulted in lower risk and rate of rescue percutaneous biliary drainage with comparable survival benefits between those who initially received PS and those who received SEMS for their MHO. As length of survival of patients with unresectable MHO is expected to continue to improve in future, it may be a quite reasonable to make the decision of initial ERBD as step-wise endoscopic approach with considerations for further plan of endoscopic revision.

## Data Availability

The datasets generated during and/or analyzed during the current study are available from the corresponding author on reasonable request.
